# Sawfly taxa (Hymenoptera, Symphyta) described by Edward Newman and Charles Healy

**DOI:** 10.3897/zookeys.398.6595

**Published:** 2014-04-04

**Authors:** Andrew D. Liston, Marko Prous

**Affiliations:** 1 Senckenberg Deutsches Entomologisches Institut, Eberswalder Str. 90, 15374 Müncheberg, Germany; 2 Department of Zoology, Institute of Ecology and Earth Sciences, University of Tartu, Vanemuise 46, 51014 Tartu, Estonia

**Keywords:** Taxonomy, Tenthredinidae, Cephidae, *Euura*, *Phylloecus*, *Hartigia*, new synonyms, new combinations

## Abstract

Type specimens of seven nominal species of sawfly described by Edward Newman and one by Charles Healy were studied. This material is housed in the Oxford University Museum of Natural History, United Kingdom. The following new synonymies are proposed (valid names in parentheses): *Hartigia* Schiødte, 1839 (*Phylloecus* Newman, 1838), *Cephus helleri* Taschenberg, 1871 (*Phylloecus faunus* Newman, 1838) and *Euura gallae* Newman, 1837 (*Euura mucronata* (Hartig, 1837)). The type species of *Euura* Newman, 1837 and *Euura* subgenus *Gemmura* E. L. Smith, 1968 belong to the same taxonomic species, *Euura mucronata* (Hartig, 1837), so that these genus group names become new synonyms. Lectotypes are designated for *Phyllotoma tormentillae* Healy, 1868, *Fenusa ianthe* Newman, 1837, *Fenusa parviceps* Newman, 1837, *Selandria pallida* Newman, 1837 and *Phylloecus faunus* Newman, 1838. 26 new combinations are proposed for species formerly placed in *Hartigia* and here transferred to *Phylloecus*, and 4 original combinations are re-instated as valid.

## Introduction

Edward Newman (1801–1876) described 24 species-group and six genus-group sawfly taxa as new to science. In many cases, the type material of these nominal taxa has apparently never been re-examined. During ongoing studies on West Palaearctic nematine sawflies (see STI Nematinae Group 2013), it became clear that clarification of the identity of *Euura gallae* Newman, 1837 is necessary. This being the type species of *Euura* Newman, 1837, the correct interpretation of the species name is required to ensure future nomenclatural stability. Through the kind assistance of the staff of the Oxford University Museum of Natural History (OUMNH), potential type specimens of several species described by Newman were located and sent to us for examination. Although only a few of these taxa belong to the Nematinae, it seems appropriate to deal here with the entire material, as well as the type series of a species described by Charles Healy (1826–1876). Newman undertook the identification of the Tenthredinidae, mostly leaf-mining species, on which Healy published several papers describing their biology.

In his introduction, Newman (1837) stated that the material referred to in that article was “in the possession of the Entomological Club”. The statement applies also to the sawflies discussed by Newman (1838), which despite its different title, is effectively a continuation of the same work. The Hymenoptera in the collection of the Entomological Club were donated in to the OUMNH in 1927 (Smith AZ 1986; J. Hogan personal communication).

## Material and methods

**Figures 1–6.**
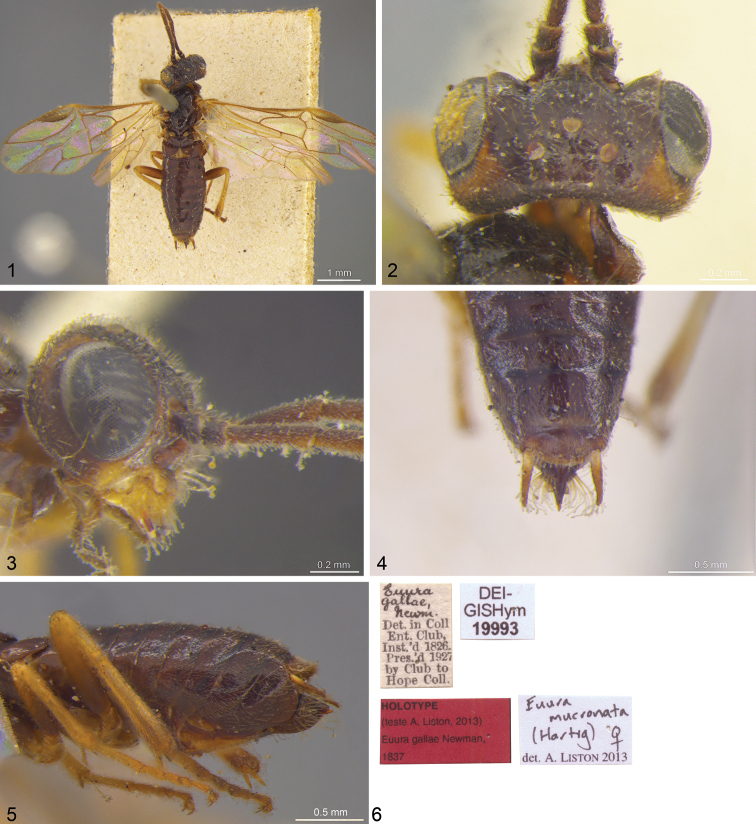
*Euura gallae* Newman, 1837; holotype **1** dorsal **2** head, dorsal **3** head, lateral **4** abdomen, dorsoapical **5** abdomen, lateral **6** labels.

**Figures 7–12.**
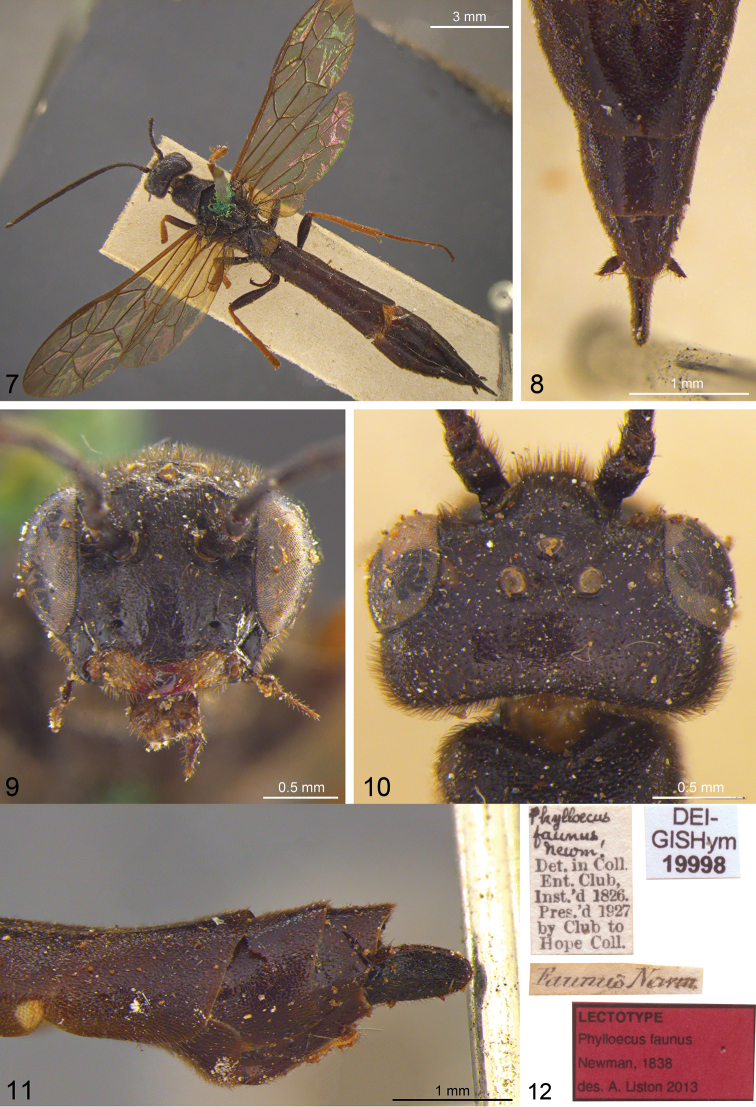
*Phylloecus faunus* Newman, 1838; lectotype. **7** dorsal **8** abdomen, dorsoapical **9** head, frontal **10** head, dorsal **11** abdomen, lateroapical **12** labels.

All specimens mentioned in this paper are deposited in the Hope Collections, Oxford University Museum of Natural History, Oxford, United Kingdom. They are all mounted in a similar way (Figs 1, 7): pinned along the dorso-ventral axis through the thorax with a short, headless pin which is carried on a small cardboard stage supported by a longer pin with a head. When the specimens were received for examination, nearly all had only a single label, with an identical printed, lower part (Figs 6, 12). At the top of this label appears the handwritten name under which the specimens stood in the collection of the Entomological Club. Although these labels are not original, they are interpreted here as representing determinations made by Newman. None of the specimens bears any data on collection locality or date on the labels or cardboard stage.

Taxa are listed in alphabetical order, under their current valid names. Complete lists of all the synonyms of species mentioned below and references to their original descriptions may be found in Taeger et al. (2010). Below are cited only the names and descriptions of taxa described by Newman or Healy, and of taxa considered here to be conspecific with the former, when the latter names are in general current use as valid.

## Results and discussion

### Euura mucronata

(Hartig, 1837)

http://species-id.net/wiki/Euura_mucronata

(Tenthredinidae)

Euura gallae Newman, January 1837: 260; sex not stated; type locality: Scotland. syn. n.
Nematus (Cryptocampus) mucronatus Hartig, March 1837: 223; ♀♂; type locality: not stated.

#### Type material examined.

*Euura gallae*. Holotype ♀, figs 1–6: “[handwritten] Euura gallae Newm. [printed] Det. in Coll. Ent. Club, Inst.’d 1826. Pres’d 1927 by Club to Hope Coll.”, “[red] Holotype (teste A. Liston, 2013) Euura gallae Newman, 1837”, “Euura mucronata (Hartig, 1837) det. Liston 2013”, “DEI-GISHym 19993”. Condition: apical three flagellomeres of both antennae and right rear tarsus missing.

#### Discussion.

Newman’s very short original description of *Euura gallae*, based on a single specimen [holotype], is impossible to identify as belonging to one of the currently recognised species. The description reads: “Euura gallae. *Nigra: antennis nigris, apice ferrugineis: pedibus pallidis*. Black: mouth yellow; antennæ rust-coloured at the tip; the legs *entirely* pale. The insect is the size of *Nematus pallipes*: the only specimen I have observed was taken by Mr. Walker, in Scotland.”

Five specimens bearing the name *Euura gallae*, all females, were found in the Hope Collections. Four of these belong to the *Euura atra* species group. They have nearly completely dark mouthparts, except for the labrum, and the femora are conspicuously black basally. They therefore do not agree with the description of the holotype. The fifth specimen (Figs 1–6) has more extensively pale mouthparts and antennae, the malar area is conspicuously pale, and the legs are almost completely pale. This specimen is identified as the holotype of *Euura gallae*.

Newman’s description of *Euura gallae* pre-dates Hartig’s of *Nematus mucronatus* by a couple of months. Article 23.9 of the International Code of Zoological Nomenclature is here applied, to reverse the precedence of the species names, because the name *Euura gallae* (nomen oblitum) has not been used as valid after 1899, and *Euura mucronata* (*Nematus mucronatus*: nomen protectum) has been used as a valid species name in [very many] more than 25 works published by more than 10 authors in the last 50 years. A list of these references is available from us on request.

The type species of *Euura* Newman, 1837 by subsequent designation of Rohwer (1911: 80) is *Euura gallae* Newman, 1837. Dalla Torre (1894: 276) listed *Euura gallae* as a valid species of *Cryptocampus* Hartig, 1837, but with a footnote “= ? *C. saliceti* (Fall.).” At this time, *Cryptocampus saliceti* (Fallén, 1808) was in use as the name of the species called *Euura mucronata* (Hartig, 1837) by most recent authors. Konow (1905b) placed *Euura gallae* as a synonym of *Cryptocampus medullarius* (Hartig, 1837). The latter is a junior subjective synonym of the species currently known as *Euura amerinae* (Linnaeus, 1758). Rohwer (1911: 94) and all subsequent authors followed Konow’s opinion, in that *gallae* was regarded as a synonym of *Euura amerinae*, or one of the subjective junior synonyms of that taxon. It is unlikely that any specialist, apart from Newman himself, has examined the holotype of *Euura gallae*. Clearly, the identity of *Euura gallae* has until now been widely misinterpreted. As a result of the new identification, the genus group name *Gemmura* E. L. Smith, 1968 (type species *Nematus mucronatus* Hartig, 1837), proposed as a subgenus of *Euura*, becomes a junior objective synonym of *Euura* Newman, sensu stricto. If in future it should be considered that recognition of subgenera within *Euura* is necessary, then a new name for the stem-galling groups would be needed. However, in our opinion there is at present neither sufficient phylogenetic support, nor a practical justification (because the genus includes too few species) for such an act. Distinction of species groups, if considered necessary, should be achieved by employing “informal” group names whose use is not regulated by the International Code of Zoological Nomenclature. Such names might, for example, be the “mucronata group” for the bud-gallers and the “atra group” for the stem-gallers.

### Fenella nigrita

Westwood, 1839

http://species-id.net/wiki/Fenella_nigrita

(Tenthredinidae)

Fenella nigrita Westwood, 1839: 54; sex not stated; type locality: not stated.
Phyllotoma tormentillae Healy, 1868: 140–141; larvae; adults reared [sex not stated] but not described; type locality: Highgate, Hornsey, Hampstead, Norwood and Croydon [parts of London].

#### Type material examined.

*Phyllotoma tormentillae*. Lectotype (**hereby designated**) ♀ [adult]: “[handwritten] Phyllotoma tormentillae, N. [printed] Det. in Coll. Ent. Club, Inst.’d 1826. Pres’d 1927 by Club to Hope Coll.”; “[red] Lectotype Phyllotoma tormentillae Healy, 1868 des. A. Liston 2013”; “Fenella nigrita Westwood, 1839 det. A. Liston 2013”, “DEI-GISHym 19994”. Condition: fair. Paralectotypes: 11♀ with same original labels, [blue] paralectotype labels, and “Fenella nigrita Westwood, 1839 det. A. Liston 2013”. One paralectotype has a large handwritten label reading “Phyllotoma Tormentillae N A complete life history of this species by Mr Healy appears in the Entomologist Vol iv p140”.

#### Discussion.

It is considered that the lectotype of *Phyllotoma tormentillae* [adult] was reared from the larvae described by Healy. The synonymy of *Phyllotoma tormentillae* with *Fenella nigrita* as given by Kirby (1882) and in various subsequent works is confirmed.

### Harpiphorus lepidus

(Klug, 1818)

http://species-id.net/wiki/Harpiphorus_lepidus

(Tenthredinidae)

Tenthredo (Emphytus) lepida Klug, 1818: 277–278; ♀♂; type locality: Germany.
Fenusa ianthe Newman, 1837: 261; sex not stated; type locality: “[..]woods of the metropolitan district[..]” (= around London).
Asticta ianthe : Newman (1838: 484), comb. n.

#### Type material examined.

*Fenusa ianthe*. Lectotype (**hereby designated**) ♀:”[handwritten] Phyllotoma”; “[handwritten] P. ianthe, Newm [printed] Det. in Coll. Ent. Club, Inst.’d 1826. Pres’d 1927 by Club to Hope Coll.”; “[red] Lectotype Fenusa ianthe Newman, 1837 des. A. Liston 2013”; “Harpiphorus lepidus (Klug, 1818) det. A. Liston 2013”, “DEI-GISHym 19995”. Condition: fair, but apical tarsomeres of all legs missing.

#### Discussion.

Although the sex of the type specimen[s] is not explicitly mentioned by Newman, the described colour pattern is found only in the female of this species. The comment “This insect appears generally distributed[..]” leads one to suppose that the description is based on more than one specimen. However, Westwood (1839: 53) wrote “This description is drawn from Mr. Newman’s typical specimen, which he has been so kind as to lend me; and of which the fore wings are unlike, the transverse nerve separating the first two submarginal cells being obliterated in one of them[..]”. The specimen here designated as lectotype possesses this abnormality (vein Rs+M is missing in the left forewing), and therefore is probably the same specimen as examined by Westwood. The synonymy of *Fenusa ianthe* with *Harpiphorus lepidus*, already adopted by Kirby (1882), is confirmed.

### Heterarthrus nemoratus

(Fallén, 1808)

http://species-id.net/wiki/Heterarthrus_nemoratus

(Tenthredinidae)

Hylotoma nemorata Fallén, 1808: 47; ♀; type locality: Sweden [according to title of work].
Fenusa parviceps Newman, 1837: 261–262; sex not stated; type locality not stated.
Druida parviceps (Newman, 1837); Newman 1838: 484.

#### Type material examined.

*Fenusa parviceps*. Lectotype (**hereby designated**) ♀:”[handwritten] Druida parviceps, Newm [printed] Det. in Coll. Ent. Club, Inst.’d 1826. Pres’d 1927 by Club to Hope Coll.”; “[red] Lectotype Fenusa parviceps Newman, 1837 des. A. Liston 2013”; “Heterarthrus nemoratus (Fallén, 1808) det. A. Liston 2013”, “DEI-GISHym 19996”. Condition: fair. Paralectotypes: 2♀ and 1 cocoon-disc with same original labels, [blue] paralectotype labels, and “Heterarthrus nemoratus (Fallén, 1808) det. A. Liston 2013”.

#### Discussion.

The synonymy of *Fenusa parviceps* with *Heterarthrus nemoratus* as proposed by Cameron (1876) and adopted in numerous subsequent works, is confirmed.

### Hoplocampa alpina

(Zetterstedt, 1838)

http://species-id.net/wiki/Hoplocampa_alpina

(Tenthredinidae)

Tenthredo alpina Zetterstedt, 1838: 339; ♀♂; type locality: “Raschstind in insula Schiervoe Nordlandiae; Gamstenstind ad Alteidet” [in northern Norway: see clarification by Greve (1986)].
Selandria pallida Newman, 1837: 262; sex not stated; type locality: not stated.

#### Type material examined.

*Selandria pallida*. Lectotype (**hereby designated**) ♀:”[handwritten] Hoplocampa pallida, Steph. [printed] Det. in Coll. Ent. Club, Inst.’d 1826. Pres’d 1927 by Club to Hope Coll.”; “[red] Lectotype Selandria pallida Newman, 1837 des. A. Liston 2013”; “Hoplocampa alpina (Zetterstedt, 1838) det. A. Liston 2013”, “DEI-GISHym 19997”. Condition: fair. Paralectotype: 1♂ with same original label, [blue] paralectotype label, and “Hoplocampa alpina (Zetterstedt, 1838) det. A. Liston 2013”.

#### Discussion.

Within *Hoplocampa*, *Selandria pallida* Newman is a junior secondary homonym of *Tenthredo pallida* Serville, 1823 (= *Hoplocampa flava* (Linnaeus, 1760): Lacourt 2000). The synonymy of *Selandria pallida* with *Hoplocampa alpina*, which has long been recognised (e.g. Kirby 1882), is confirmed.

### Phylloecus

Newman, 1838

http://species-id.net/wiki/Phylloecus

(Cephidae)

Phylloecus Newman, 1838: 485–486.
Phylloecus : Rohwer 1911; type species designated as *Phylloecus faunus* Newman, 1838; placed as synonym of *Janus* Stephens, 1829.
Hartigia Schiødte, 1839: 331–332, 347, 370. Boie 1855; type species designated as *Astatus satyrus* Panzer, 1801 [= *Phylloecus niger* (Harris, [1779])]. syn. n.

#### Discussion.

*Phylloecus faunus* was stated by Abe and Smith (1991) to have been designated by monotypy as the type species of *Phylloecus* Newman, 1838. This is not so, because Newman (1838, p. 486) ends his discussion on his new genus with the words “[..] but it seemed to me that the division containing *Faunus*,&c. is equally distinct, and therefore I would submit the propriety of raising these also, to the rank of a genus, under the name *Phylloecus*”. His foregoing text makes it clear that at least *Cephus satyrus* (Panzer, 1801) (a junior synonym of *Hartigia nigra* (M. Harris, [1779]) was thus considered also to belong to *Phylloecus*. Rohwer (1911) interpreted this correctly and accordingly designated *Phylloecus faunus* as type species. However, Rohwer (1911, p. 94 [index], under the names *cynosbati* and *faunus*) makes it clear that he regarded *Phylloecus faunus* as conspecific with *Janus cynosbati* (Linnaeus, 1758) (= *Janus femoratus* (Curtis, 1830): see Blank et al. (2009) on nomenclature). From Newman’s description and subsequent discussion it is evident that his concept of *Phylloecus* corresponds closely with that of what in recent years has been called *Hartigia*, and this correct interpretation was followed by various authors during the 19th Century. The lectotype of *Phylloecus faunus* belongs to the species recently known as *Hartigia helleri* (Taschenberg, 1871) (see below, under *Phylloecus faunus*). Benson (1951) and Pagliano and Scaramozzino (1990) treated *Hartigia* and *Phylloecus* as synonymous, but did not use the latter as the valid name. On the other hand, the misinterpretation of *Phylloecus* as *Janus* also has a long history, which can be traced back at least to Kirby (1882), and in recent years this wrong synonymy has become universally accepted. The International Code of Zoological Nomenclature (ICZN 1999) unfortunately provides no opportunity of maintaining the name *Hartigia* in precedence over *Phylloecus*, because the use of *Phylloecus* as a valid name after 1899, by for example Marchand (1902) and Richter von Binnenthal (1903), precludes the application of Article 23.9. (reversal of precedence). Neither are the species of *Phylloecus* of such economic, scientific or cultural importance that an application to the Commission to conserve the name *Hartigia* seems likely to achieve success, although some species are of rather minor significance to growers of soft fruit and ornamental roses in North America (Smith DR 1986), and *Phylloecus faunus* has been considered for use in the biological control of *Rubus* in Australia (e.g. Bruzzese 1982; as *Hartigia albomaculatus*). As a result of the new synonymy, the following species names are either newly transferred to *Phylloecus* (comb. n.) or the original name combinations are re-instated as valid (comb. rev.). New combinations are followed in parentheses by the original combination of the species group name. Only the nominal species which were considered to be valid by Taeger et al. (2010) are listed:

*Phylloecus agilis* (F. Smith, 1874), comb. n. (*Cephus agilis*)

*Phylloecus albotegularis* (Wei & Nie, 1996), comb. n. (*Hartigia albotegularis*)

*Phylloecus algiricus* André, 1881 comb. rev.

*Phylloecus bicinctus* Provancher, 1875 comb. rev.

*Phylloecus cheni* (Wei & Nie, 1999), comb. n. (*Hartigia cheni*)

*Phylloecus coreanus* (Takeuchi, 1938), comb. n. (*Hartigia coreana*)

*Phylloecus cowichanus* (Ries, 1937), comb. n. (*Hartigia cowichana*)

*Phylloecus elevatus* (Maa, 1944), comb. n. (*Hartigia elevata*)

*Phylloecus epigonus* (Zhelochovtsev, 1961), comb. n. (*Hartigia epigona*)

*Phylloecus etorofensis* (Takeuchi, 1955), comb. n. (*Hartigia etorofensis*)

*Phylloecus fasciatus* (Cresson, 1880), comb. n. (*Cephus fasciatus*)

*Phylloecus faunus* Newman, 1838, comb. rev.

*Phylloecus kamijoi* (Shinohara, 1999), comb. n. (*Hartigia kamijoi*)

*Phylloecus linearis* (Schrank, 1781), comb. n. (*Tenthredo linearis*)

*Phylloecus mexicanus* (Guerin, [1844]), comb. n. (*Cephus mexicanus*)

*Phylloecus minutus* (Wei & Nie, 1997), comb. n. (*Hartigia minuta*)

*Phylloecus niger* (M. Harris, [1779]), comb. n. (*Sirex niger*)

*Phylloecus nigratus* (Dovnar-Zapolskij, 1931), comb. n. (*Pachycephus nigratus*)

*Phylloecus nigritus* (Forsius, 1918), comb. n. (*Macrocephus nigritus*)

*Phylloecus nigrotibialis* (Wei & Nie, *1977*), comb. n. (*Hartigia nigrotibialis*)

*Phylloecus pyrrha* (Zhelochovtsev, 1968), comb. n. (*Hartigia pyrrha*) [Zhelochovtsev gives no etymology for this species name. It is here considered to be a noun, the name of a figure in Greek mythology]

*Phylloecus riesi* (D. R. Smith, 1986), comb. n. (*Hartigia riesi*)

*Phylloecus sibiricola* Jakovlev, 1891 comb. rev.

*Phylloecus simulator* (Kokujev, 1910), comb. n. (*Macrocephus simulator*)

*Phylloecus stackelbergi* (Gussakovskij, 1945), comb. n. (*Hissarocephus stackelbergi*)

*Phylloecus stigmaticalis* (Wei & Nie, 1996), comb. n. (*Hartigia stigmaticalis*)

*Phylloecus trimaculatus* (Say, 1824), comb. n. (*Cephus trimaculatus*)

*Phylloecus viator* (F. Smith, 1874), comb. n. (*Cephus viator*)

*Phylloecus xanthostoma* (Eversmann, 1847), comb. n. (*Cephus xanthostoma*)

*Phylloecus zhengi* (Wei & Nie, 1996), comb. n. (*Hartigia zhengi*)

### Phylloecus faunus

Newman, 1838 spec. rev.

http://species-id.net/wiki/Phylloecus_faunus

(Cephidae)

Phylloecus faunus Newman, 1838: 485–486; ♀♂; type locality: “in the vicinity of London”. Note: *faunus* is a noun; the name of a Roman deity.
Cephus helleri Taschenberg, 1871: 305–306; ♀; type locality: Insula Lesina [Island of Hvar, Croatia]. syn. n.

#### Type material examined.

*Phylloecus faunus*. Lectotype (**hereby designated**) ♀, Figs 7–12. “[handwritten] Phylloecus faunus, Newm. [printed] Det. in Coll. Ent. Club, Inst.’d 1826. Pres’d 1927 by Club to Hope Coll.”; “[handwritten] Faunus Newm.”; “[red] Lectotype Phylloecus faunus Newman, 1838 des. A. Liston 2013”; “Hartigia faunus (Newman, 1838) det. A. Liston 2013”. Condition: missing most of right antennal flagellum, most tarsi except right middle and rear; abdomen after tergum 5 glued to specimen.

#### Discussion.

[see also under *Phylloecus*, above]. Newman refers to a syntype series of three specimens of *Phylloecus faunus*: “Two specimens of this insect have been taken by Mr. Ingall, and one by Mr. Stephens”. The single specimen examined agrees well with the brief description. Most taxonomic works and catalogues (e.g. Konow 1905a; Taeger et al. 2010) have until now placed *Phylloecus faunus* as a synonym of *Janus cynosbati* (Linnaeus, 1758), although it should have been apparent from several characters described or discussed by Newman (1838), that these are not conspecific. The mistaken synonymy was possibly first published by Kirby (1882).

Although the name *faunus* has not to the best of our knowledge been used as valid after 1899, neither has the name *helleri* been sufficiently used (in 21 publications by 27 authors including co-authors) as valid in the last fifty years to satisfy the conditions of Article 23.9 (reversal of precedence) of the International Code of Zoological Nomenclature (ICZN 1999). A list of these references is available from us on request. The lectotype of *Phylloecus faunus* agrees in all important points with the characterisation of *Hartigia helleri* by Jansen (1998). Quinlan (1970) identified a second female specimen in the Natural History Museum, London, which should be regarded as a paralectotype of *Phylloecus faunus*, as *Hartigia albomaculatus* [sic!], noted that it bore a label “faunas” [presumably in reality *faunus*] and mentioned that no reliable information is available on where it was caught. One might doubt the reliability of Newman’s statement that the types of *Phylloecus faunus* were collected around London, because under its synonyms *Hartigia albomaculata* and *Hartigia helleri* no evidence for the presence of this species in the British Isles has been published, and because neither of the two type specimens still in existence bears any explicit label data referring to the collection locality. However, an occurrence in the London area, at least historically, seems not unlikely. Chevin (1993) presented several records from northern France, under the name *Hartigia albomaculata*, and later (Chevin and Chevin 2007) recorded *Hartigia helleri* from the Département de la Manche, not far from the Channel coast. It is concluded that *Phylloecus faunus* should be used as the valid name of the species referred to in recent years first as *Hartigia albomaculata* (or *Hartigia albomaculatus*, misspelling) and latterly as *Hartigia helleri*, and that after weighing up the evidence, the type locality of *Phylloecus faunus* can be accepted as being in the area of London.

### Specimens without type status

Amongst the specimens borrowed for examination were also the following Tenthredinidae, apparently identified by Newman. None of these specimens is considered to be a type.

1♂ *Euura atra* (Jurine, 1807), det. A. Liston, with handwritten superscript on the printed label “Euura cynips, Newm.” and the following additional labels: “[printed] 1 Cynips Newm.”, “[handwritten / blue paper] Euura roboris Newman”. Remarks: The colouration of this specimen (completely black antennae, femora basally black) does not fit Newman’s (1837: 260) very short original description of the male of *Euura cynips*. The specimen therefore cannot be considered to belong to the type series of *Euura cynips*. Newman (1869: 319) wrote that “*Euura cynips* produces the familiar gall to be found almost everywhere on the leaves of the crack willow (*Salix fragilis*): this gall is of an oblong form, and protrudes equally from both surfaces of the leaf; it is usually of a red tint on the upper surface [..]”. This statement clearly refers to the gall of *Pontania proxima* (Serville, 1823), but having been published more than thirty years after the description of *Euura cynips*, it cannot be used as an argument for interpreting the name as a synonym of *Pontania proxima*. Based on the inadequate original description, Liston et al. (2006) treated *Euura cynips* as a synonym of *Euura testaceipes* (Brischke, 1883) and as a nomen oblitum. This treatment should be maintained.

1♀ *Heterarthrus ochropoda* (Klug, 1818), det. A. Liston, with handwritten superscript on the printed label “Druida populi”. Remarks: No publication has been located in which the name “Druida populi” is used.

2♀ *Pontania proxima* (Serville, 1823), det. A. Liston, with handwritten superscript on the printed label “Euura roboris, Newm.” Remarks: No publication has been located in which the name “Euura roboris” is used. Newman (1869) did make a name *Euura quercus* available (Taeger et al. 2010), by publishing a seven-word description of a gall on oak that he supposed to have been caused by a sawfly. Whether this has anything to do with “E. roboris”, a name possibly indicating a relationship with *Quercus robur*, cannot at present be answered.

## Supplementary Material

XML Treatment for Euura mucronata

XML Treatment for Fenella nigrita

XML Treatment for Harpiphorus lepidus

XML Treatment for Heterarthrus nemoratus

XML Treatment for Hoplocampa alpina

XML Treatment for Phylloecus

XML Treatment for Phylloecus faunus

## References

[B1] Abe M Smith DR (1991) The Genus-group Names of Symphyta (Hymenoptera) and Their Type Species. Esakia 31: 1–115

[B2] André E (1881) Species des Hyménoptères d‘Europe & d‘Algérie. L’Auteur, Beaune (Côte-d’Or), 1[1879–1882](10): 485–564, catalogue 57–70.

[B3] Benson RB (1951) Hymenoptera, Symphyta. Handbooks for the Identification of British Insects 6(2a): 1–49

[B4] Blank SM Taeger A Liston AD Smith DR Rasnitsyn AP Shinohara A Heidemaa M Viitasaari M (2009) Studies toward a World Catalog of Symphyta (Hymenoptera). Zootaxa 2254: 1–96

[B5] Boie F (1855) Beobachtungen und Bemerkungen. Stettiner entomologische Zeitung 16: 48–51

[B6] Brischke CGA (1883) Beobachtungen über die Arten der Blatt- und Holzwespen von C. G. A. Brischke, Hauptlehrer a. D. in Langfuhr und Dr. Gustav Zaddach weiland Professor in Königsberg. Zweite Abtheilung. Schriften der Naturforschenden Gesellschaft in Danzig, N. S., 5[1881–1883](4): 201–328

[B7] Bruzzese E (1982) The host specificity of Hartigia albomaculatus (Hym., Cephidae) and its potential effectiveness in the biological control of European blackberry. Entomophaga 27(3): 335–342 doi: 10.1007/BF02374817

[B8] Cameron P (1876) A Monographic revision of the British species of Phyllotoma. Proceedings (& Transactions) of the Natural History Society of Glasgow 2[1869–1875]: 315–321

[B9] Chevin H (1993) Hartigia albomacula (Stein) espece souvent confondue avec Hartigia nigra (Harris) (Hymenoptera, Cephidae). L‘Entomologiste. Revue d‘Amateurs 49(6): 273–276

[B10] Chevin H Chevin S (2007) Inventaire des Hyménoptères Symphytes (Tenthrèdes) du Département de la Manche. Cahiers des Naturalistes, Bulletin des Naturalistes Parisiens 56(1–2): 1–22

[B11] Cresson ET (1880) Descriptions of new North American Hymenoptera in the collection of the American Entomological Society. Transactions of the American Entomological Society 8: 1–52

[B12] Curtis J (1830) British Entomology; being illustrations and descriptions of the genera of Insects found in Great Britain and Ireland: containing Coloured Figures from Nature of the most rare and beautiful species, and in many instances of the plants upon which they are found. Published by the Author, London, 7(parts 73–84, plates 290–337), 2 pp. text to each plate

[B13] Dalla Torre CG de (1894) Catalogus Hymenopterorum hucusque descriptorum systematicus et synonymicus. Vol. 1: Tenthredinidae incl. Uroceridae (Phyllophaga & Xylophaga) Sumptibus Guilelmi Engelmann, Lipsiae, [6]+VIII+459 pp

[B14] Dovnar-Zapolskij DP (1931) Cephiden Studien (Hymenoptera, Chalastogastra) (I. Beitrag). Ezhegodnik Zoologicheskogo Muzeja 32: 37–49

[B15] Eversmann E (1847) Fauna hymenopterologica volgo-uralensis exhibens Hymenopterorum species quas in provinciis Volgam fluvium inter et montes Uralenses sitis observavit et nunc descripsit. Bulletin de la Société Impériale des Naturalistes de Moscou 20(1): 3–68

[B16] Fallén CF (1808) Försok till uppställning och beskrifning å de i Sverige fundne Arter af Insect-Slägtet Tenthredo Linn. Kongliga Vetenskaps Academiens nya Handlingar 29(1): 39–64

[B17] Forsius R (1918) Über einige von Bequaert in Nordafrika gesammelte Tenthredinoiden. Översigt af Finska Vetenskaps Societetens Förhandlingar 60[1917–1918](13): 1–11

[B18] Greve L (1986) Gamstind / Gamstenstind og Raschtind. To insektlokaliteter fra Zetterstedts tid som i dag ikke finnes pa norske kartverk. Insekt-Nytt. Medlemsblad for Norsk entomologisk forening 11(1): 12–13

[B19] Guérin-Méneville FÉ [1844] Neuvième Ordre. - Hyménoptères In: Guérin-Méneville FÉ (1829–1844) Iconographie du Règne animal de G. Cuvier, ou représentation d’après nature, de l’une des espèces les plus remarquables et souvent non encore figurées, de chaque genre d’animaux, avec un text descritif mis au courant de la science. Ouvrage pouvant servir d’atlas a tous les traités de zoologie. Baillière, Paris, 7(50 livr.), 7: 398–466

[B20] Gussakovskij VV (1945) A new genus of Cephidae (Hymenoptera) from Tadjikistan. Doklady Akademii Nauk SSSR 48(7): 530–531

[B21] Harris M (1776-[1780]) An exposition of English insects, with curious observations and remarks, wherein each insect is particularly described; its Parts and Properties considered; the different Sexes distinguished, and the Natural History faithfully related. The whole illustrated with copper plates, drawn, engraved and coloured by the Author Moses Harris. [Une exposition des insectes Anglois, avec des observationes et des remarques curieuses, dans lesquelles chaque insecte est particuliérement décrit; ses parties et ses propriétes sont considerées; leurs sexes distingués, et leur histoire naturelle fidellement récitée. Le tout enrichie des tailles douces, dessinées, gravées, et colorées par l’auteur, Moise Harris.] (In English and French). White & Robson, London, VIII+166+[4] pp

[B22] Hartig T (1837) Die Aderflügler Deutschlands mit besonderer Berücksichtigung ihres Larvenzustandes und ihres Wirkens in Wäldern und Gärten für Entomologen, Wald- und Gartenbesitzer. Die Familien der Blattwespen und Holzwespen nebst einer allgemeinen Einleitung zur Naturgeschichte der Hymenopteren. 1 Haude & Spener, Berlin, xiv+416 pp. & VIII plates

[B23] Healy C (1868) A Life-history of Phyllotoma Tormentillae. The Entomologist 4(58): 140–141

[B24] ICZN [International Commission on Zoological Nomenclature] (1999) International code of zoological nomenclature. Fourth Edition The International Trust for Zoological Nomenclature, London, 306 pp.

[B25] Jakovlev A (1891) Diagnoses Tenthredinidarum novarum ex Rossia Europaea, Sibiria, Asia Media et confinum. Trudy Russkago Entomologitscheskago Obschtschestva v S. Peterburge 26[1892]: 1–62 (Separatum).

[B26] Jansen E (1998) Die Gattung Hartigia Schiødte, 1838 in Europa (Hymenoptera: Cephidae). In: Taeger A Blank SM (Eds) Pflanzenwespen Deutschlands (Hymenoptera, Symphyta). Kommentierte Bestandsaufnahme. Goecke & Evers, Keltern 301–318

[B27] Kirby WF (1882) List of Hymenoptera with Descriptions and Figures of the Typical Specimens in the British Museum. 1. Tenthredinidae and Siricidae Printed by order of the trustees, London, 450 pp.

[B28] Klug F (1818) Die Blattwespen nach ihren Gattungen und Arten zusammengestellt. Der Gesellschaft Naturforschender Freunde zu Berlin Magazin für die neuesten Entdeckungen in der gesamten Naturkunde 8(1814)(4): 273–307

[B29] Kokujev N (1910) O rasprostraneni v Rossii pereponchatokrylykh nasekomykh, iz podsemejjstva Cephini Konow (Hymenoptera Chalastogastra Konow), i opisanie novykh vidov. [On the distribution of the Hymenoptera of the subfamily Cephini Konow (Hymenoptera Chalastogastra Konow) in Russia, and the description of new species.] Russkoe Entomologicheskoe obozrenie 10(3): 127–139 [In Russian and Latin]

[B30] Konow FW (1905a) Hymenoptera. Fam. Lydidae. Genera Insectorum 27: 1–27

[B31] Konow FW (1905b) Hymenoptera. Fam. Tenthredinidae. Genera Insectorum 29: 1–176

[B32] Lacourt J (2000) Liste des espèces de la famille des Tenthredinidae décrites par J. G. Audinet-Serville, en Mai 1823 et par A. L. M. Le Peletier Comte de Saint-Fargeau, en Août 1823, avec désignation de lectotypes (Hymenoptera, Symphyta). Revue française d’Entomologie (N. S.) 22(2–3): 77–108

[B33] Linnaeus C (1758) Systema Naturae, per regna tria naturae secundum classes, ordines, genera, species cum characteribus, differentiis, synonymis, locis. 1 Editio Decima, Reformata Laurentius Salvius, Holmiae, 824 pp.

[B34] Linnaeus C (1760) Fauna Svecica sistens animalia Sveciae regni: Mammalia, Aves, Amphibia, Pisces, Insecta, Vermes. Distributa per classes & ordines, genera & species, cum differentiis specierum, synonymis auctorum, nominibus incolarum, locis natalium, descriptionibus insectorum. Editio altera, auctior Laurentius Salvius, Stockholmiae, 578 pp.

[B35] Liston AD Taeger A Blank SM (2006) Comments on European Sawflies (Hymenoptera: Symphyta). In: Blank SM Schmidt S Taeger A (Eds) Recent Sawfly Research: Synthesis and Prospects. Goecke & Evers, Keltern, 245–263

[B36] Maa T (1944) Novelties of Chinese Hymenoptera Chalastogastra. Biological Bulletin of Fukien Christian University 4: 33–60

[B37] Marchand E (1902) Inventaire des Tenthrédonides ou Mouches à scie (Hymenoptera - Chalastogastra) recueillies aux environs de Nantes suivi de notices sur quelques espèces particulièrement nuisibles. Bulletin de la Société des Sciences Naturelles de l’Ouest de la France, 2e sér., 2(3–4): 233–296

[B38] Newman E (1837) Notes on Tenthredinina. The Entomological Magazine 4[1836–1837](3): 258–263

[B39] Newman E (1838) Entomological notes. The Entomological Magazine 5[1837–1838](2, 4, 5): 483–500

[B40] Newman E (1869) Concerning phytophagous Hymenoptera whose Larvae are concealed. The Entomologist 4: 318–321

[B41] Pagliano G Scaramozzino PL (1990) Elenco dei Generi di Hymenoptera del Mondo. Memorie della Società Entomologica Italiana 68/Suppl. 122[1989](1): 1–210

[B42] Panzer GWF [1801] Faunae Insectorum Germanicae initia oder Deutschlands Insecten. Felssecker, Nürnberg, 8[1801–1804](85): 24 col. plates & 24 pp

[B43] Provancher L (1875) Les Urocerides de Québec. Le Naturaliste Canadien 7: 368–376

[B44] Quinlan J (1970) The identity of Hartigia albomaculatus (J. P. E. F. Stein) (Hymenoptera: Tenthredinidae). The Entomologist 103: 304–306

[B45] Richter von Binnenthal F (1903) Die Rosenschädlinge aus dem Tierreiche, deren wirksame Abwehr und Bekämpfung. Ein Ratgeber für die gärtnerische Praxis. Verlagsbuchhandlung Eugen Ulmer, Stuttgart, X+392 pp

[B46] Ries DT (1937) A New Species of Sawfly, Hartigia cowichana, from Canada (Cephidae-Hymenoptera). Entomological News 48(3): 82–83

[B47] Rohwer SA (1911) Technical papers on miscellaneous forest insects. II. The genotypes of the sawflies and woodwasps, or the superfamily Tenthredinoidea. Technical series / US Department of Agriculture, Bureau of Entomology 20: 69–109

[B48] Say T (1824) Appendix [Insecta] In: Keating WH (Ed) Narrative of an expedition to the source of St. Peter’s River, Lake Winnepeek, Lake of the Woods, &c. &c. performed in the year 1823, by order of the Hon. J. C. Calhoun, Secretary of War, under the command of Stephen H. Long, Major U.S.T.E. 2 Carey & Lea, Philadelphia, 268–378

[B49] Schiødte JC (1839) Beretning om Resultaterne af en i Sommeren 1838 foretagen entomologisk Undersøgelse af det sydlige Sjaelland, en Deel af Laaland, og Bornholm. Naturhistorisk Tidsskrift 2[1838–1839](4): 309–394

[B50] Schrank F von P (1781) Enumeratio Insectorum Austriae indigenorum. E. Klett et Franck, Augustae Vindelicorum, [23]+550 pp

[B51] Serville AJG (1823) Hyménoptères Térébrans Porte-scie. In: Vieillot P Desmarest AG Durcrotay de Blainville H Audinet-Serville A Le Peletier de Saint Fargeau A Walckenaer CA (Eds) Faune Française, ou Histoire naturelle, générale et particulière, des animaux qui se trouvent en France, constamment ou passagèrement, à la surface du sol, dans les eaux qui le baignent, et dans le littoral des mers qui le bornent. 7–8 Levrault, Paris, 1–96

[B52] Shinohara A (1999) A study on Stem Boring Sawflies (Hymenoptera, Cephidae) of the Tribe Hartigiini from Japan and Korea. Japanese Journal of Systematic Entomology 5(1): 61–77

[B53] Smith AZ (1986) A History of the Hope Entomological Collections in the University Museum Oxford with lists of Archives and Collections. Clarendon Press, Oxford, 172 pp.

[B54] Smith DR (1986) The berry and rose stem-borers of the genus Hartigia in North America (Hymenoptera: Cephidae). Transactions of the American Entomological Society 112: 129–145

[B55] Smith EL (1968) Biosystematics and Morphology of Symphyta. I. Stem-Galling Euura of the California Region, and a New Female Genitalic Nomenclature. Annals of the Entomological Society of America 61(6): 1389–1407

[B56] Smith F (1874) Descriptions of new species of Tenthredinidae, Ichneumonidae, Chrysididae, Formicidae &c. of Japan. Transactions of the Entomological Society of London for the Year 1874(3): 373–409

[B57] Stephens JF (1829) A Systematic Catalogue of British Insects: being an attempt to arrange all the hitherto discovered indigenous insects in accordance with their natural affinities. Containing also references to every English writer on entomology, etc[...]. 1 Baldwin & Cradock, London, xxxiv+416 pp

[B58] STI Nematinae Group (STING); Liston AD Prous M Blank SM Taeger A Heibo E Vårdal H (2013) Revising (half) the Nematinae (Tenthredinidae) of the West Palaearctic. Hamuli. The Newsletter of the International Society of Hymenopterists 4(2): 1–5

[B59] Taeger A Blank SM Liston AD (2010) World Catalog of Symphyta (Hymenoptera). Zootaxa 2580: 1–1064

[B60] Takeuchi K (1938) A systematic study on the suborder Symphyta (Hymenoptera) of the Japanese Empire (I). Tenthredo. Acta Entomologica 2(2): 173–229

[B61] Takeuchi K (1955) Sawflies of the Kurile Islands (I). Insecta Matsumurana 19(1–2): 9–22

[B62] Taschenberg EL (1871) Einige neue südeuropäische Hymenoptera. Zeitschrift für die gesammten Naturwissenschaften 38: 305–311

[B63] Wei M Nie H (1996) Studies of Chinese Cephidae III. The Genus Hartigia Schiodte (Hymenoptera: Cephidae: Hartigiini). Journal of Central South Forestry University 16(3): 9–14 [In Chinese, abstract in English]

[B64] Wei M Nie H (1997) Studies on Chinese Cephidae: Notes on the species of stem-sawfly deposited in Zhejiang Agricultural University (Hymenoptera: Cephidae). Journal of Zhejiang Agricultural University 23(5): 523–528 [In Chinese, abstract in English]

[B65] Wei M Nie H (1999) A new species of Cephidae from the south slope of Mt. Funiu (Hymenoptera: Cephomorpha). In: Shen X Pei H (Eds) Insects of the mountains Funiu and Dabie regions. The Fauna and Taxonomy of Insects in Henan, Vol. 4 China Agricultural Science and Technology Press, Beijing, 136–137 [In Chinese, abstract in English]

[B66] Westwood JO (1839) Synopsis of the Genera of British Insects. In: Westwood JO (1838–1840) An Introduction to the modern Classification of Insects; founded on the natural habits and corresponding organisation of the different families. Longman, Orme, Brown, Green & Longmans, London, 49–80

[B67] Zetterstedt JW (1838) Ordo IV. Hymenoptera. In: Zetterstedt JW (Ed) Insecta Lapponica descripta. Sectio Secunda L. Voss, Lipsiae, 326–358

[B68] Zhelochovtsev AN (1961) Novye i maloizvestnye pilil’shhiki (Hymenoptera, Symphita [sic!]) Tjan’-Shanja. [New and little known sawflies (Hymenoptera, Symphyta) from Tian-Shan.] Sbornik trudov Zoologicheskogo Muzeja MGU 8: 117–138

[B69] Zhelochovtsev AN (1968) Novye vidy Symphyta (Hymenoptera) fauny SSSR. [New species of Symphyta (Hymenoptera) of the fauna of the USSR.] Sbornik trudov Zoologicheskogo Muzeja MGU 11: 47–56

